# Physical properties of the tunic in the pinkish-brown salp *Pegea confoederata* (Tunicata: Thaliacea)

**DOI:** 10.1186/s40851-018-0091-1

**Published:** 2018-04-12

**Authors:** Daisuke Sakai, Hiroshi Kakiuchida, Jun Nishikawa, Euichi Hirose

**Affiliations:** 1 School of Regional Innovation and Social Design Engineering, Kitami Institute of Technology, Koen-cho, Kitami, Hokkaido 090-8507 Japan; 2 Structural Materials Research Institute, National Institute of Advanced Industrial Science and Technology (AIST), Moriyama, Nagoya, Aichi 463-8560 Japan; 3 Department of Marine Biology, School of Marine Science and Technology, Tokai University, Orido, Shimizu, Shizuoka, 424-8610 Japan; 4 Faculty of Science, University of the Ryukyus, Nishihara, Okinawa, 903-0213 Japan

**Keywords:** Ultrastructure, Hardness, Transparency, Light reflection; spectroscopic ellipsometry; nipple array; rigorous coupled wave analysis (RCWA), Salps, *Pegea*

## Abstract

**Background:**

Invisibility in the water column is a crucial strategy for gelatinous zooplanktons in avoiding detection by visual predators, especially for animals distributed in the euphotic zone during the daytime; i.e., surface dwellers that do not undergo diel vertical migration. Salps, a member of the subphylum Tunicata (Urochordata), usually have a transparent body that is entirely covered with a cellulosic matrix, called the tunic. Some non-migrator species are known to exhibit a nano-scale nipple array on the tunic surface. However, the physical properties of the salp tunic has been poorly investigated, except for *Thetys vagina*, in which the tunic was expected to show low reflectance based on the refractive index of the tunic. *Pegea confoederata* is a non-vertical migrant salp showing pinkish-brown body. We measured the hardness, water content, absorption spectra, and refractive index of its tunic to evaluate its fragility and visibility.

**Results:**

There are nipple-like protuberances about 80 nm high on the surface of the tunic in *P. confoederata*. The tunic is very soft; the maximum force to pierce the tunic with a steel rod (1 mm diameter) was < 1 N. The water content of the tunic was > 95%. The absorption spectra of the tunic had no prominent peaks in the wavelength range of 280–800 nm, indicating the tunic is nearly transparent. The difference in refractive indices between tunic and seawater was estimated as 0.002–0.015 at 589 nm. Rigorous coupled wave analyses (RCWA) of light reflection based on 3-dimensional models supported an anti-reflective effect of the nipple array on the tunic surface, which was estimated to vary slightly depending on the forms and the arrangement patterns of nipple-like protuberances in an array.

**Conclusions:**

The tunic of *P. confoederata* is very soft and contains more water than those of sessile tunicates (ascidians). Based on the refractive index of the tunic, light reflection is expected to be very low, making this salp’s tunic barely visible in water column. Our results suggest that the nipple array may produce an anti-reflective effect.

**Electronic supplementary material:**

The online version of this article (10.1186/s40851-018-0091-1) contains supplementary material, which is available to authorized users.

## Background

Many gelatinous zooplankton, such as jellyfish and salps, have transparent bodies, which may be beneficial for avoiding visual predators. Salps (Salpida: Thaliacea) belong to subphylum Tunicata (= Urochordata), the sister group of Vertebrata. Tunicates always exhibit cellulosic tissue secreted from epidermis; this is known as the tunic in Ascidiacea and Thaliacea and their house in Appendicularia [1–4]. In salps, a gelatinous, transparent tunic covers the entire body, and there are no prominent peaks in the absorbance spectra of the tunic within 280–800 nm light, i.e., visible light, ultraviolet (UV) A and B, in *Thalia rhomboides* and *T. vagina* [5, 6]. Moreover, our Abbe-refractometric and ellipsometric measurements indicated that the difference in refractive indices was estimated to be 0.03 or less between seawater and the tunic surface of *T. vagina*, as a result, reflectance on the tunic surface at incident angles of 80° or less is < 10% [6]. Accordingly, salps are expected to be rarely visible under water, and this is consistent with our field observations of salps underwater.

The nipple array is a nano-structure comprised of nipple-shaped protrusions of about 100 nm in height. This structure was originally found on the surface of the compound eyes of moths and shows anti-reflective property, the so-called “moth-eye effect,” by forming a refractive index gradient [7, 8]. Similar structures have been described in some aquatic invertebrates including ascidians [9] and salps [3]. Interestingly, salps possessing nipple arrays occur in the shallow layers of the water column throughout the day, whereas the other salps are distributed in deeper and darker layers during the day and come up to shallow layers at night, i.e. undergo diel vertical migration [10, 11]. This may imply a relationship between the absence or presence of a nipple array and light and/or visibility of the salps in euphotic zone.

**Fig. 1:**
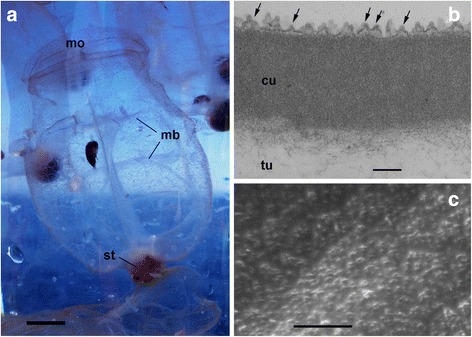
Zooids and tunic of *Pegea confoederata*. **a** Aggregate zooids alive onboard. **b** Cross section of the tunic cuticle (TEM). **c** Protrusions on the tunic surface (SEM). Arrows indicate some of the protrusions on the tunic cuticle (cu). mb, muscle band; mo, mouth; st, stomach; tu, tunic matrix. Scale bars: 1 cm in (**a)**, 0.2 μm in (**b)**, 1 μm in (**c)**

*Pegea confoederata* is a salp often distributed in the euphotic zone during the daytime, i.e. they are non-vertical migrants, and often has pinkish/brownish body (Fig. 1a). The rather conspicuous body color of this species may suggest that the reduction of visibility against the potential visual predator for them may not be important for the survival of this species. In the present study, we examined the detailed physical properties of the tunic of *P. confoederata*, i.e., hardness, light absorption, and refractive index. Based on the refractive index and the surface structure of the tunic, we simulated light reflectance on the 3-dimensional models of the tunic surface to determine how the salps appear in a bright water column.

## Methods

### Animals

Chains of the salp *Pegea confoederata*, floating at the surface water, were collected at Suruga Bay, Japan using a scoop net during the daytime on November 5th, 2016 during the research cruise of RV Hokuto of Tokai University (Fig. 1a). After collection, they were transferred into sample bottles and brought to the laboratory alive. Three aggregate zooids (30–40 mm in length) were frozen and stored at − 80 °C until use for the measurement of physical properties. Additionally, some solitary zooids released from the live aggregate zooids were fixed with 2.5% glutaraldehyde in seawater onboard and stored at 4 °C for microscopy. The frozen specimens were thawed at room temperature, and section of the tunic was excised from the middle of the body. Mantle tissues were manually removed from the tunic unless otherwise noted. It was impossible for us to measure the physical properties of fresh specimens, because the salps were collected by chance and the measurement was carried out in the measurement room a long distance from the collection site. Accordingly, we measured the physical properties of the frozen-thawed specimens that were at risk of damage during freezing and thawing. However, the measured values are expected to be similar to those of fresh specimens. For instance, the refractive indices measured by an Abbe refractometer were similar between the fresh and frozen–thawed tunic in an ascidian *Rhopalaea* sp. [6], and no significant difference in hardness was observed between fresh and frozen–thawed tunic in an ascidian *Halocynthia roretzi* (Hirose, unpublished).

### Microscopy

Tunic pieces were cut from the glutaraldehyde-fixed salps using a razor blade. The specimens were rinsed with 0.45 M sucrose and 0.1 M cacodylate buffer (pH 7.5) and post-fixed with 1% osmium tetroxide in a 0.1 M cacodylate buffer (pH 7.5) at 4°C for 1.5 h. Specimens were dehydrated through an ethanol series. For scanning electron microscope (SEM), the dehydrated specimens (two individuals) were immersed in *t*-butanol, freeze-dried in a *t*-butanol freeze dryer (VFD-21S; Vacuum Device), sputter-coated with gold-palladium, and examined under a scanning electron microscope (JSM-6060LV; JEOL) at 15 kV. For transmission electron microscope (TEM), the dehydrated specimens (two individuals) were cleared with *n*-butyl glycidyl ether and embedded in an epoxy resin (Epon 812, TAAB Laboratories). Thin sections at approximately right angle to the surface were stained with uranyl acetate and lead citrate, and examined in a transmission electron microscope (JEM1011, JEOL) at 80 kV. The heights and intervals of the cuticular protrusions were measured from electron micrographs.

### Hardness and water content of tunics

Three frozen salps were thawed at room temperature. A tunic piece was cut from the middle part of the body. After briefly blotting excess water on a paper towel, the tunic specimens were weighted (wet weight), and thickness of the specimen was measured with a vernier caliper. Then, the specimen was sandwiched with two acrylic plates (5 mm thick) with a hole (3 mm diameter). A pin attachment (TP-20, IMADA Co., Ltd., Toyohashi, Japan) was mounted to a digital force gauge DS2-5 N (IMADA Co., Ltd.) that was fixed on a lever test stand FCA-50 N (IMADA Co., Ltd.). The pin was a steel rod (1 mm diameter) with a flat tip. Pulling down the lever of the test stand, the force gauge with the pin came down perpendicularly and the pin pierced the tunic specimen between the acryl plates through the holes. The maximum force to pierce the specimen was recorded at five separate points randomly selected within each specimen, and the median was regarded as the hardness of each specimen. The measured values of hardness vary depending on the method, such as the shape of the attachment of the force gauge, and thus, the values are comparable with those obtained by the same method. After the measurement above, each specimen was dried in an oven at 60 °C for 3 days and weighed (dry weight). The water content (%) in the tunic specimens was obtained from a ratio of dry weight to wet weight.

### Absorption spectra of tunics

The tunics of *P. confoederata* were mounted on the wall of a quartz cell with a small amount of seawater, and the 280–800 nm absorption spectra were recorded at 2-nm intervals with a U-4100 spectrophotometer (Hitachi High-Technologies Corp., Tokyo, Japan). The absorption spectra were compared with those of transparent tunics of an ascidian *Rhopalaea* sp. and a salp *Thetys vagina* [6]. As a reference for a tunic containing UV-absorbing substances, we also used the absorption spectrum of *Diplosoma virens*, a colonial ascidian containing mycosporine-like amino acids [12].

### Refractometry with an Abbe refractometer

Refractive indices (nD) of seven tunic specimens were measured using an Abbe refractometer (NAR-1 T SOLID: Atago Co., Ltd., Tokyo, Japan) at 24.5–25.0 °C with LED light approximating to the wavelength of D-line (ca. 589 nm). The outer surface of the tunic, i.e., cuticular side, faced the prism of the refractometer. We also examined refractive index of artificial seawater (Marine Art SF-1: Osakayakken Co., Ltd.) for five times, and obtained the difference between the tunic and seawater.

### Ellipsometry

We examined one tunic specimen with mantle (Sample 1) and three specimens without mantle (Samples 2–4). The tunics of *P. confoederata* were spread on a glass plate, and the wavelength dispersion of refractive index was determined in the 320–1000 nm range, using a spectroscopic ellipsometer (M2000, J. A. Woolam Co. Inc.) that analyzes polarization properties of light reflected from the surface of materials. Focusing probes were installed in this ellipsometric system to find a flat surface of the specimens placed on a flat plate. The measurements were carried out at 70°, 75°, and 80° (incident angles) to minimize the effects of backside reflection, which is unfavorable for precise analyses. A diffusive light absorbing foil (specular reflectance less than 0.2%; Metal Velvet™, Acktar Ltd.) was used as plate to reduce stray light from backside reflection through the transparent specimens, since stray light may influence the ellipsometric analysis. Backside reflection is defined as the number of secondary reflections produced between front- and backside surfaces in the transparent substrate, and it was estimated to be very small, i.e., 0.05 or less in the present measurements.

The ellipsometric parameters *Ψ* and *Δ* were measured and were analyzed assuming simple optical structure, such as a transparent bulk sample with flat front- and backside surfaces. The wavelength dispersion of refractive index was assumed to follow the Cauchy model [13]. The optical model for ellipsometric analysis was evaluated by MSE (mean squared error) between the measured and theoretical values for *Ψ* and *Δ.* The refractive indices (*n*) at wavelengths (λ) between 320 and 1000 nm are calculated from the Cauchy dispersion formula with a three-term form:

$$ n=\mathrm{a}+\mathrm{b}/\lambda 2+\mathrm{c}/\lambda 4 $$

Based on the parameters measured with the spectroscopic ellipsometer, we obtained the coefficient values (a, b and c), backside reflection, and fit errors. Here, fit errors indicate the range in which there is no effective gaps between the measured and theoretical values for *Ψ* and *Δ*.

### Simulation of light reflection with and without a nipple array

The light reflection was calculated at the border between the medium (seawater) and matrix (tunic) with or without the nipple array with RCWA using DiffractMOD3.2 software (RSoft Design Group, Inc., Ossining, NY, USA). In the simulation, seawater was assumed to be 35‰ at 25 °C, and the refractive index was estimated following the equation [14] that was valid from 400 nm to 700 nm light. The differences in refractive indices between the tunic and seawater were assumed to be 0.01, according to the estimates of refractive indices measured at 589 nm with the refractometer and the ellipsometer. Polarized lights were used for this simulation, i.e., TE and TM wave. Based on the electron microscope observations, we examined three forms of 3-dimensional models of nipple array (i.e., cone, pillar, and two-tier) arranged in grid and honeycomb patterns, respectively (see Results). Parameters for the simulation were defined as follows: wavelength, 589 nm; incident angle, 0–89.9°. The simulation was also carried out at 400 nm in some conditions.

## Results

### Fine structures of the tunic surface

The outermost part of the tunic is an electron-dense layer of about 0.7 μm in thickness, i.e., cuticle, and minute protrusions form a nipple array on the surface of the tunic cuticle (Fig. 1b, c). In the sections, the protrusion appears to be a simple cone or a two-tier cone. There are some variations in the average height of each protrusion, whereas average interval between the apical tips of the protrusions is about 0.1 μm (Table 1).

**Table 1:** Average size of the nipple array

Sample	Numbers	Height ± SD (μm)	Interval ± SD (μm)^*^
A	21	0.081 ± 0.018	0.1 ± 0.024
B	21	0.079 ± 0.011	0.101 ± 0.029
C	24	0.062 ± 0.011	0.1 ± 0.026

^*^Intervals between the apical tips of neighbor protrusions

### Hardness and water content

As shown in Table 2, the hardness of the tunic was less than 1 N, and water content was more than 95% of the tunic. Thickness of the tunic specimens was about 0.1 mm.

**Table 2:** Hardness and water content of *Pegea confoederata* tunic

Sample	Hardness (N)^*^	Water content (%)
I	0.447	96.1
II	0.504	98.3
III	0.388	97.5

^*^Median of the five measurements of the maximum force to pierce the tunic with a stainless rod (1 mm diameter)

### Absorption spectra

**Fig. 2:**
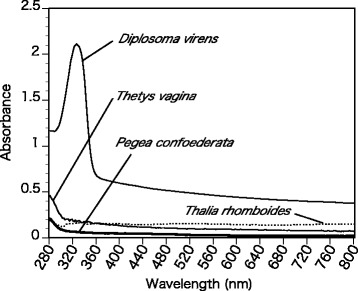
Absorption spectrum of the tunic of *Pegea confoederata* with the spectra of *Diplosoma virens*, *Rhopalaea* sp. and *Thetys vagina*. The spectra of *P. confoederata*, *T. vagina* and *Rhopalaea* sp. have no prominent peaks, whereas the spectrum of *D. virens* has an absorption peak due to UV-absorbing substances

In *P. confoederata*, the absorption spectrum of the tunic has no specific absorption peaks in the range of visible light (400–800 nm), UV-A (325–400 nm), or UV-B (280–315 nm); the same is true in *T. vagina* and *Rhopalaea* sp. (Fig. 2). In *D. virens* tunic containing UV-absorbing substances, the absorption spectra had a prominent absorption peak at around 320 nm (Fig. 2).

### Refractive index

The refractive indices of the tunic measured by the Abbe refractometer ranged from 1.3414 to 1.3430 (*n* = 7; average, 1.3421; s.d., ± 0.0006) at D-line light (λ = 589 nm), while the refractive indices of artificial seawater ranged from 1.3388 to 1.3394 (*n* = 5; average, 1.3392; s.d., ± 0.0002). The difference between the tunic and seawater was about 0.003.

Mean squared errors (MSEs) in ellipsometric analyses indicate the difference in ellipsometric parameters (*Ψ* and ***Δ***) between the experimental and theoretical curves. We obtained MSEs, coefficients of Cauchy dispersion formula, backside reflection, and fit errors for each sample (Table 3). Ellipsometric parameter *Ψ* (left) and ***Δ*** (right) of the tunic are shown in Additional file 1. While MSE values were < 10 in all samples, MSE of the specimen with a mantle tissue (Sample-1) was nearly double that of the other specimens in which mantle was removed.

**Table 3:** Coefficients in the ellipsometric analysis for *Pegea confoederata* tunic

Specimen	MSE	Coefficient (± Fit error)			Backside reflection (± Fit error)
		a	b	c	
1^*^	9.261	1.3363 (± 0.00242)	0.000351 (± 0.00138)	0.00043 (± 0.000201)	0.012349 (± 0.00794)
2	3.742	1.3402 (± 0.00112)	0.004796 (± 0.000651)	−0.000061 (± 0.000096)	0.052867 (± 0.00335)
3	4.557	1.3276 (± 0.00129)	0.008344 (± 0.00073)	−0.0004 (± 0.000106)	0 (± 0.00412)
4	5.658	1.3732 (± 0.00171)	0.002983 (± 0.000971)	−0.00027 (± 0.000141)	0.01324 (± 0.00499)

^*^Tunic specimen with mantle tissue

**Fig. 3:**
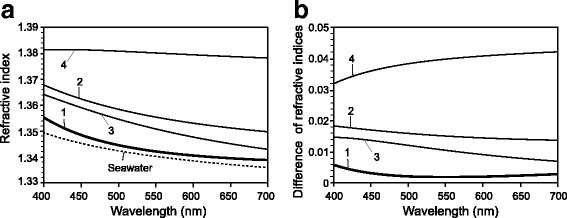
Refractive indices estimated by ellipsometry. **a** Refractive indices of seawater (dotted line), the tunic of *Pegea confoederata* with mantle (1, thick line) and the tunic without mantle (2–3, thin lines). The refractive index of seawater (35 ‰, 25 °C) is calculated from the equation [14] that is valid from 400 nm to 700 nm. **b** Difference in refractive indices between the tunic specimens and seawater

Fig. 3 shows the refractive indices estimated by ellipsometric calculation in wavelength range from 400 to 700 nm. The refractive index of Sample-4 is prominently larger than those of the other samples (Sample 1–3). When the result for Sample-4 (1.3796 at 589 nm) was excluded as an irregular case, the refractive indices of the tunic and the difference between the tunic and seawater ranged 1.3409–1.3535 and 0.002–0.0147 at 589 nm, respectively.

### Simulation of the light reflection on the nipple array

**Fig. 4:**
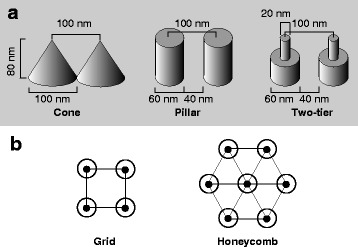
**a** Simplified 3-dimensional models of the nipple array for the simulation of light reflection. **b** Grid and honeycomb patterns of the arrays for the simulation

The reflection of 589-nm light was calculated at the border between seawater and tunic with or without the nipple array by rigorous coupled wave analyses (RCWA), assuming the difference in refractive indices was 0.01. Based on the ultrastructural observation, we simplified the nipple array structures for simulation as three forms of 3-dimensional models, i.e., cone, pillar, and two-tier (Fig. 4a) arranged in grid and honeycomb patterns, respectively (Fig. 4b).

**Fig. 5:**
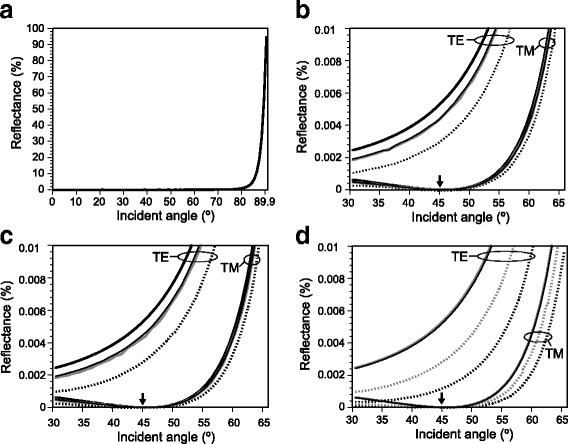
Simulation of light reflectance (%) with rigorous coupled wave analysis (RCWA) on the flat surface and 3-dimensional models of nipple array (i.e., cone, pillar, and two-tier) at various incident angles. The difference in refractive index between the tunic and seawater was assumed to be 0.01. **a** Reflectance of 589-nm light of TE wave on the flat surface. **b** and **c**, Enlargement of the reflectance curves on the flat surface and on the nipple array arranged in grid pattern (**b**) and honeycomb pattern (**c**). Lines in **b** and **c**: thick line, flat surface; thin line, cone; broken line, pillar; gray line, two-tier. **d** Reflectance of 400-nm (black) and 589-nm (gray) light on the flat surface and on the pillar-model arranged in honeycomb pattern. Line in **d**: solid line, flat surface; broken line, pillar

The difference in refractive indices was so small that light reflection occurred rarely when the incident angle was below 80°. Reflectance reached 1% at about 80° and at about 81° on the flat surface for transverse electric wave (TE wave) and transverse magnetic wave (TM wave), respectively. Although Fig. 5a shows the reflectance curves of TE wave on the flat surface, it is impossible to recognize the difference between TE and TM wave as well as the anti-reflective effect of the nipple arrays at this scale of the graph. The enlargements (Fig. 5b, c) clearly show the difference in reflectance between TE and TM wave and the anti-reflective effect that is variable among the models of nipple arrays. For instance, the reflectance of the incident light at 30° on the array of the pillars was about 40% of the reflectance on the flat surface (Fig. 5b, c). The reflectance at the same incident angle always had a relation flat > cone ≥ two-tier > pillar in both arrays of the grid and honeycomb patterns. In reflection of TM wave, the reflectance curve had a minimum at around 45° due to Brewster’s angle (arrows in Fig. 5b, c). The difference in reflectance between grid and honeycomb patterns was also small, but the tendencies were different among the models. Details are shown in Additional file 2.

At shorter wavelength (400 nm), tendencies of the reflectance curves were quite similar to those at 589 nm; the reflectance at the same incident angle always has a relation flat > cone ≥ two-tier > pillar in both arrays of the grid and honeycomb patterns. The reflectance at 400 nm was always smaller than that at 589 nm in any combinations of the nipple array models and patterns, when the difference in refractive indices between the tunic and seawater was assumed the same with 589 nm, i.e., 0.01. The difference in reflectance between 400-nm and 589-nm light was the largest on the pillar forms arranged in honeycomb pattern, while the reflectance curve on the flat surface at 400 nm was almost overlapped with that at 589 nm (Fig. 5d).

## Discussion

Tunic cuticle, the outermost, electron-dense layer of the tunic, often forms an array of protrusions of about 100 nm in height. In ascidians, the nipple arrays were found in many species of particular taxa [9], implying that nipple arrays have an important function and have been preserved phylogenetically. In salps, nipple array has been described in *Thalia democratica*, *T. orientalis*, *T. rhomboides*, *Thetys vagina*, and *P. confoederata*, while it was not found in *Iasis zonaria*, *Metcalfina hexagona*, and *Salpa fusiformis* [3, 5, 15, 16]. Among salp species we have examined so far, nipple array has been exclusively in the species distributed in the shallow layers of the water column even in daytime. The nipple array in salps may thus function under bright conditions. The nipple arrays on the tunic cuticle usually consist of simple, nipple-like protrusions. In *P. confoederata*, the protrusions are 60–80 nm in height and they often appear to be two-tier cones. Difference in shape of the protrusions may engender differences in physical properties of the tunic.

The hardness of the *P. confoederata* tunic was less than 1 N, revealing that the salps are very soft prey for the predators. In ascidian tunics, our preliminary measurements in the same method are > 30 N in a leathery tunic of *Halocynthia roretzi*, about 20 N in a cartilaginous tunic of *Styela plicata*, and about 2 N in a gelatinous tunic of *Ciona savignyi* (Hirose, unpublished). The water content of the tunic was > 95% in the present species, and this value is larger than some ascidian tunics so far reported: about 81% in *Halocynthia aurantium* [17], 71.8% in *Molgula impura* and 88% in *Styela partita* [18]. Whereas the tunics are generally leathery or cartilaginous in *Halocynthia* and *Styela* species and gelatinous in *Molgula* species, the dataset is too small to discuss possible relations between hardness and water content.

The apparent transparency of the *P. confoederata* tunic is consistent with the absorption spectra having no prominent absorption peaks in the range of 280–800 nm (Fig. 2), as well as the spectra of transparent tunics of *T. vagina*, *Thalia rhomboides*, and *Rhopalaea* sp. [5, 6]. The present specimens were frozen and thawed before the measurements in the present study, and hence, the possibility remains that molecules involved in light protection, such as ultraviolet-absorbing substances, were lost during the thawing process.

In the measurement using an Abbe refractometer at D-line (λ = 589 nm), the refractive indices of the *P. confoederata* tunic is about 1.342 and about 0.003 greater than seawater. This value is very similar to the refractive indices of the transparent tunics of an ascidian *Rhopalaea* sp. (about 1.344 in fresh specimens; about 1.343 in frozen–thawed specimens) and another salp *T. vagina* (1.342 in frozen–thawed specimens) [6]. As we discussed in the previous study, Abbe refractometer measurements would underestimate refractive index, owing to the seawater between the tunic surface and the prism [6].

In the ellipsometric analysis, MSE between the measured and theoretical values for *Ψ* and *Δ* was < 10 in all four samples, suggesting that the fitting model here was appropriate for the estimation of refractive index (see also Additional file 1). The MSE of Sample-1 was markedly larger than those of the others, and the transparent mantle tissue exclusively remained in Sample-1 possibly caused the larger MSE. Among the estimations of refractive indices of the four samples, the refractive index of Sample-4 was prominently greater than those of the other samples. It is possible that errors occurred in the measurement of Sample-4. Excluding the results of Sample-4, the refractive indices of the three samples ranged from 1.3409 to 1.3535 at 589 nm; these values are similar to those measured with the Abbe refractometer (1.3421 in average) and markedly smaller than the refractive indices of an alternative salp tunic (*Thetys vagina*: 1.368) and an ascidian tunic (*Rhopalaea* sp.: 1.364), estimated with ellipsometry [6]. There are a number of potential causes for errors in measurements by both Abbe refractometer and ellipsometer: seawater between the prism and the specimen may cause an underestimation in the measurement with the Abbe refractometer, and water evaporation from the specimens during the measurement may cause overestimation in the ellipsometry because increased salt concentration results in a greater refractive index [6]. We expect that the true refractive index of *P. confoederata* tunic is not far from the values obtained here. The tunic of *P. confoederata* was softer and much thinner than the tunics of *Rhopalaea* sp. and *T. vagina*, and this may cause the interspecific differences of the refractive index. Furthermore, since the tunic specimens of the present species were well spread as flat sheets on a plate, we could easily locate a flat surface of the specimens and carry out the measurement in a short time. As a result, the measurement errors due to the evaporation of water from the specimen would be smaller in the present study. In any case, the refractive index of *P. confoederata* is estimated to be very similar to that of seawater; the difference between tunic and seawater is 0.002–0.015.

According to the ellipsometric analysis, the refractive index of the tunic is always larger than seawater in a wavelength range from 400 to 700 nm, i.e. visible range. When incident light come from a medium of higher refractive index to a medium of lower refractive index, the light of incident angle at the critical angle or larger is totally reflected at the border of the two media. Needless to say, the occurrence of total reflection may highlight the contour of the body and is unfavorable for transparent organisms. In order to minimize light reflection in water column, the refractive indices of the tissues should be very similar to the refractive index of the ambient media (seawater or water), taking something into consideration of the potential occurrence of total reflection. The refractive index of seawater and water is variable to some extent due to salinity, temperature, and so on.

Using RCWA, we carried out the simulation of the light reflection on the flat surface and on the 3-dimensional models of nipple array. In a previous study, we adopted 2-dimensional models of nipple array comprised of a half circle on a rectangle [6]. In the 3-dimensional simulation here, we built three forms and arranged them in two patterns to analyze the effect of forms and arrangements on the reflectance. Our simulation validated anti-reflective effect of the three models of nipple array (Fig. 5b, c). However, the difference in refractive indices between seawater and the tunic of *P. confoederata* is so small that the effect was also limited; the maximum reduction of reflectance was less than 0.2 points. The anti-reflective effect was variable among the forms (i.e., cone, pillar and two-tier) and the pattern of arrangement (i.e., grid and honeycomb). In *P. confoederata*, the difference in forms and arrangement causes only small differences, because the reflectance itself is small. The forms and arrangement would cause large difference in reflectance for land organisms in which the difference in refractive indices is considerably large on the border between air and the body surface. For instance, the difference in refractive indices is 0.5 or larger between air and chitinous tissue.

Although the reflectance curve of 400-nm light on the flat surface was almost overlapped with the curve of 589-nm light, the reflectance at 400 nm was always smaller than the reflectance at 589 nm on the nipple array models (Fig. 5d). This indicates that anti-reflective effect of the nipple array is stronger when the wavelength of light is shorter. In the present simulation, we assumed the difference in refractive indices between the tunic and seawater was 0.01. However, according to the ellipsometric analysis, the difference tended to be larger when the wavelength was shorter (Fig. 3b), resulting larger reflectance of the light of shorter wavelength on the flat surface. On the other hand, anti-reflective effect of the nipple array is greater when the difference in refractive indices is larger. For instance, the difference in refractive indices between the Sample-3 and seawater was 0.0095 at 589 nm and 0.0149 at 400 nm, according to our ellipsometric analysis (Fig. 3b). Moreover, the present result indicated that the anti-reflective effect was greater at a shorter wavelength, even if the refractive index difference was the same. When the difference in refractive indices was assumed to be 0.01 at 589 nm and 0.015 at 400 nm respectively, the reflectance at 400 nm was calculated to be considerably larger than the reflectance at 589 nm on the flat surface. Interestingly, the reflectance on the nipple array (pillar, honeycomb) at 400 nm is similar to that at 589 nm (Additional file 3). Therefore, the nipple array may cancel out the increase of reflectance due to the shorter wavelength.

The pillar model always has the smallest reflectance among the three forms of the models, although the ultrastructure of nipple array appears more similar to the cone or two-tier than the pillar. This indicates that the form of nipple array is not optimized for anti-reflection in *P. confoederata*, as well as the other nipple arrays so far reported in salps [5, 6, 9, 16]. Cone (or two-tier) may be more structurally robust than pillar-shaped nipple array. It is also possible that the tunicates are not able to produce pillar-shaped protuberances on the tunic surface, although the mechanism of the biosynthesis of nipple array remains unresolved.

## Conclusion

The tunic of *P. confoederata* is almost transparent in the wavelength range of 280–800 nm and the difference in refractive indices between tunic and seawater is estimated as only 0.002–0.015 at 589 nm. These features indicate that the salp tunic is barely visible in the water column. The present results raise a question whether the presence of nipple array is functional to camouflage the salp in a water column or not. Since the small difference in refractive indices between the tunic and seawater resulted in small reflectance even on the flat surface, the anti-reflective effect of the nipple array may be unremarkable in Fig. 5a. However, closer observations revealed considerable reduction of reflectance particularly at lower angles of incidence where the reflectance on the flat surface is also very small (Fig. 5b, c). The nipple array may serve additional and/or alternative functions that are indispensable for the survival of the diurnal salp species. In this case, nipple array needs to have a suitable shape for the functions, and this may be a reason why the shape is not optimized for anti-reflection. Besides the anti-reflection function, multiple functions have been reported for nipple array, such as suppression of bubble adhesion [19], inhibition of cell spreading [20, 21], and reduction of friction [22], and these functions may be related with buoyancy control, defense against parasites, and swimming of the salps, respectively.

## Additional files

Additional file 1 Ellipsometric parameter *Ψ* (left) and ***Δ*** (right) of the tunic of *Pegea confoederata*. (PDF 677 kb)

Additional file 2 Reflectance of 589-nm light on the nipple array models arranged in the grid pattern and honeycomb pattern. (PDF 252 kb)

Additional file 3 Offset of larger reflectance at shorter wavelength by greater anti-reflective effect of the nipple array. (PDF 395 kb)

## Abbreviations

MSE: Mean squared error
RCWA: Rigorous coupled wave analysis
RV: Research vehicle
SEM: Scanning electron microscope
TE wave: Transverse electric wave
TEM: Transmission electron microscope
TM wave: Transverse magnetic wave
UV: Ultraviolet
