# Supplementary material for: Physical properties of the tunic in the pinkish-brown salp Pegea confoederata (Tunicata: Thaliacea)
Source: Zoological Lett. 2018 Apr 12;4:7. doi: 10.1186/s40851-018-0091-1 (PMC5896079; doi:10.1186/s40851-018-0091-1)

Supplementary Figure S1

TITLE: Physical properties of the tunic in the pinkish-brown salp *Pegea confoederata* (Tunicata: Thaliacea)
AUTHOR: Daisuke Sakai, Hiroshi Kakiuchida, Jun Nishikawa & Euichi Hirose

Ellipsometric parameter Ψ (left) and Δ (right) of the tunic of Sample-1.

Red lines indicate the fitted curve, and dots indicate the measured values at 70° (green), 75° (blue), and 80° (violet).

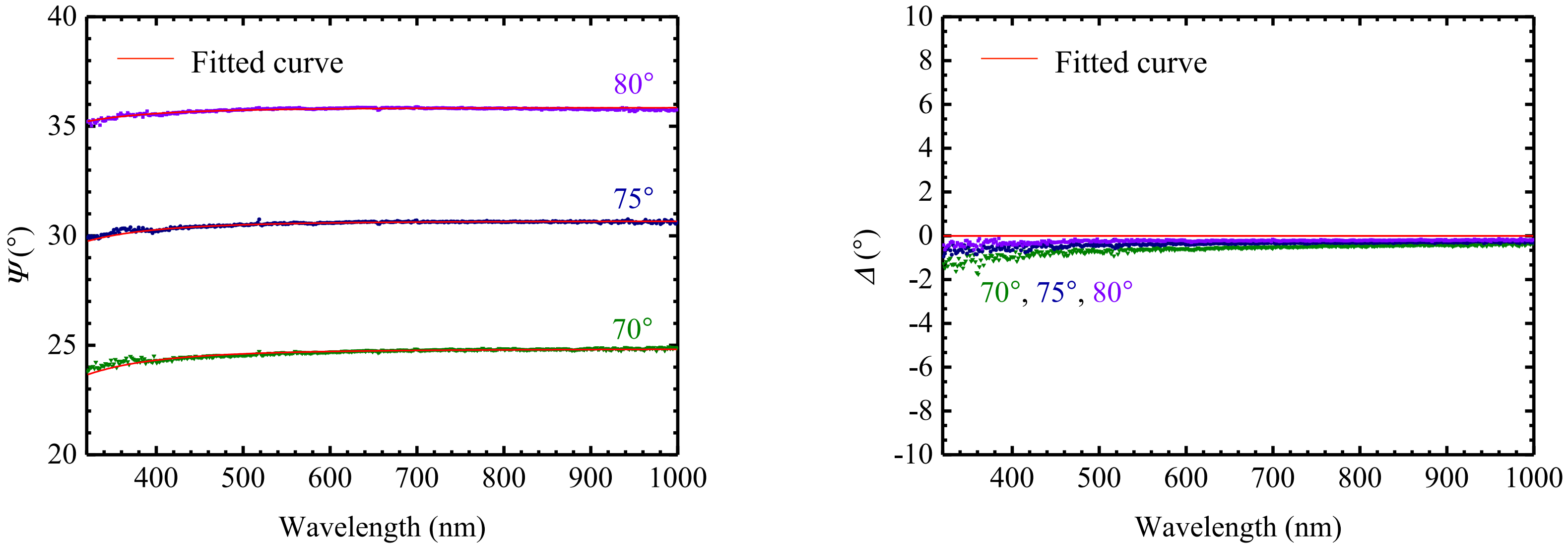

Ellipsometric parameter *Ψ* (left) and *Δ* (right) of the tunic of Sample-2.

Red lines indicate the fitted curve, and dots indicate the measured values at 70° (green), 75° (blue), and 80° (violet).

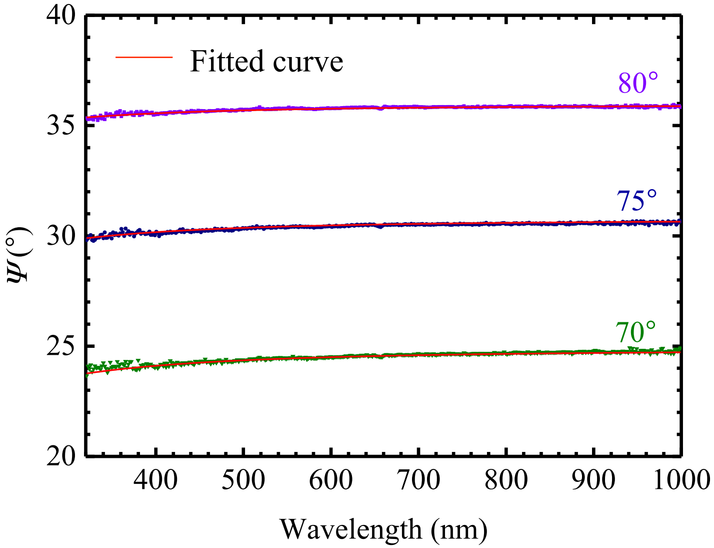

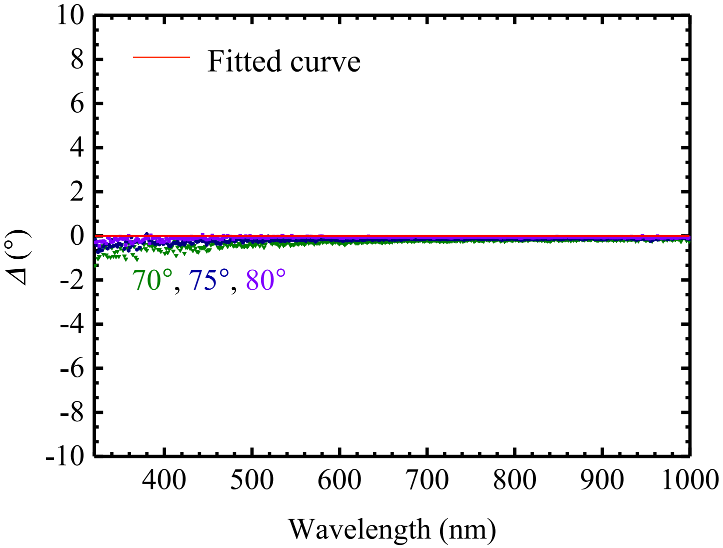

Ellipsometric parameter *Ψ* (left) and *Δ* (right) of the tunic of Sample-3.

Red lines indicate the fitted curve, and dots indicate the measured values at 70° (green), 75° (blue), and 80° (violet).

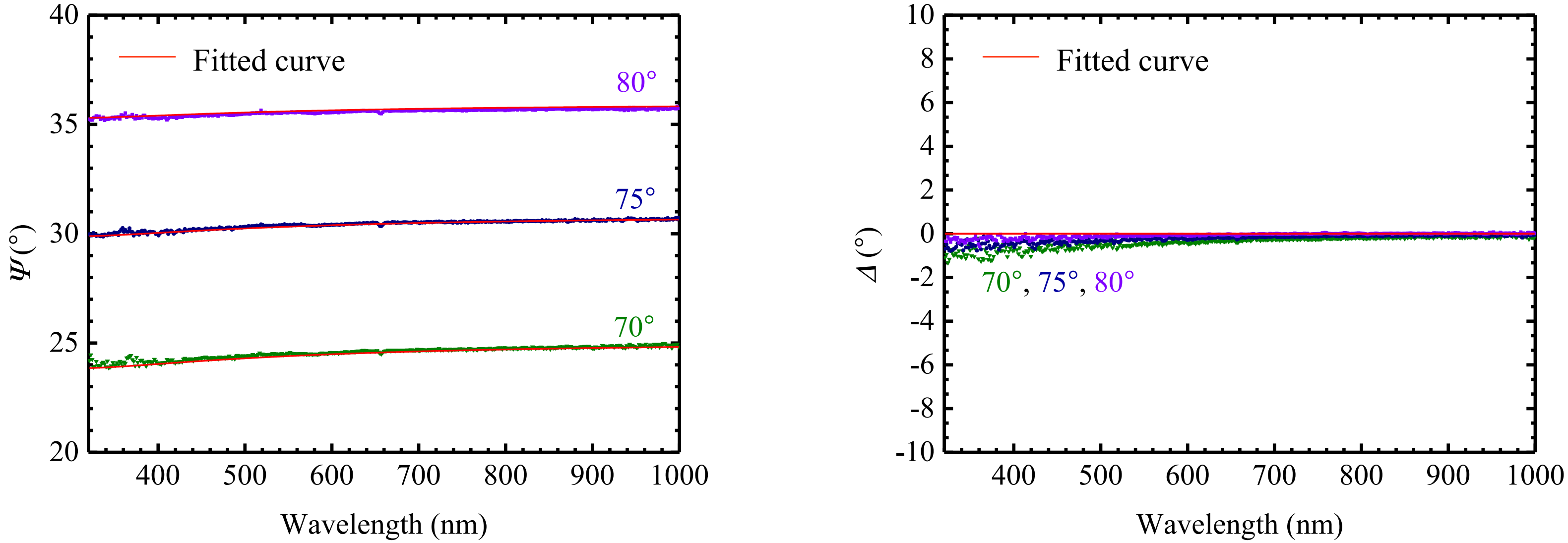

Ellipsometric parameter *Ψ* (left) and *Δ* (right) of the tunic of Sample-4.

Red lines indicate the fitted curve, and dots indicate the measured values at 70° (green), 75° (blue), and 80° (violet).

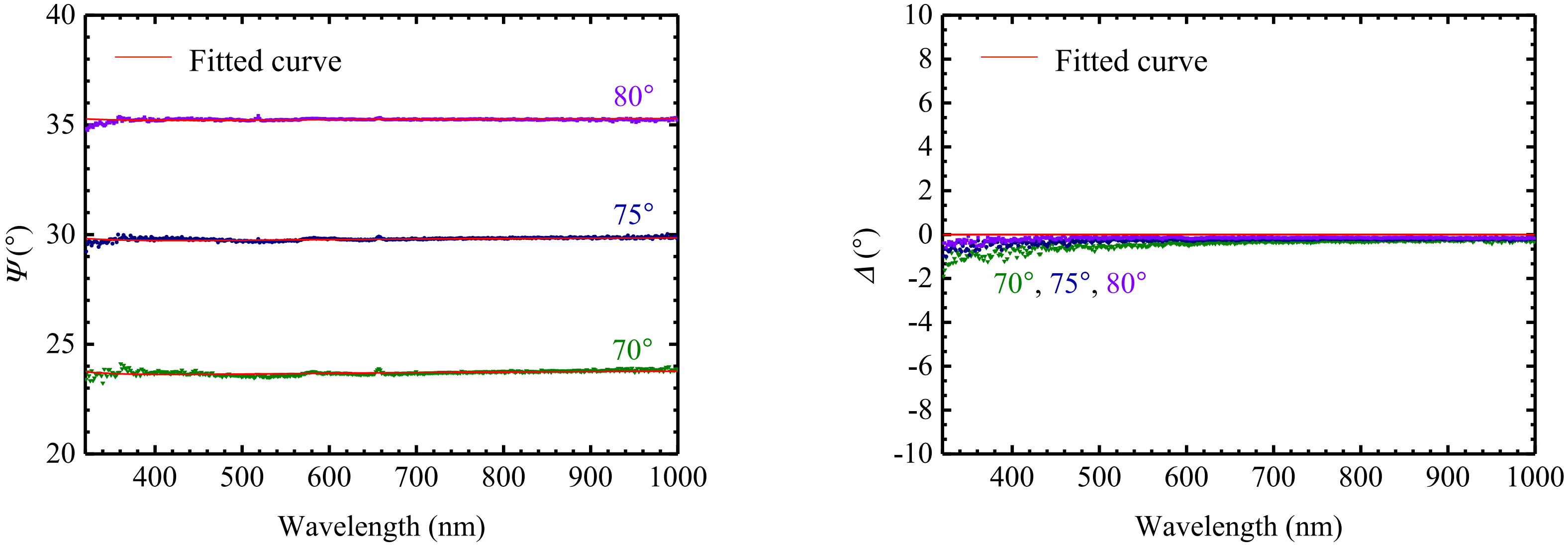

Supplement: Supplementary file 1 — Ellipsometric parameter Ψ (left) and Δ (right) of the tunic of Pegea confoederata. (PDF 677 kb) [file 40851_2018_91_MOESM1_ESM.pdf]
