# Supplementary material for: Physical properties of the tunic in the pinkish-brown salp Pegea confoederata (Tunicata: Thaliacea)
Source: Zoological Lett. 2018 Apr 12;4:7. doi: 10.1186/s40851-018-0091-1 (PMC5896079; doi:10.1186/s40851-018-0091-1)

Supplementary Figure S2

TITLE: Physical properties of the tunic in the pinkish-brown salp *Pegea confoederata* (Tunicata: Thaliacea)
AUTHOR: Daisuke Sakai, Hiroshi Kakiuchida, Jun Nishikawa & Euichi Hirose

Reflectance of 589-nm light on the nipple array models arranged in the grid pattern and honeycomb pattern

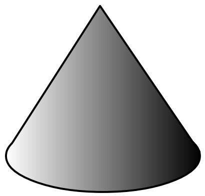

Cone-model

Solid line: TE wave
Broken line: TM wave
Red: grid pattern
Purple: honeycomb pattern

In the cone model, the reflectance was smaller on the grid pattern than the reflectance on the honeycomb pattern in the most range of the incident angle, and the difference between the patterns was very small found (< 0.003 points for TE wave and < 0.002 points for TM wave).

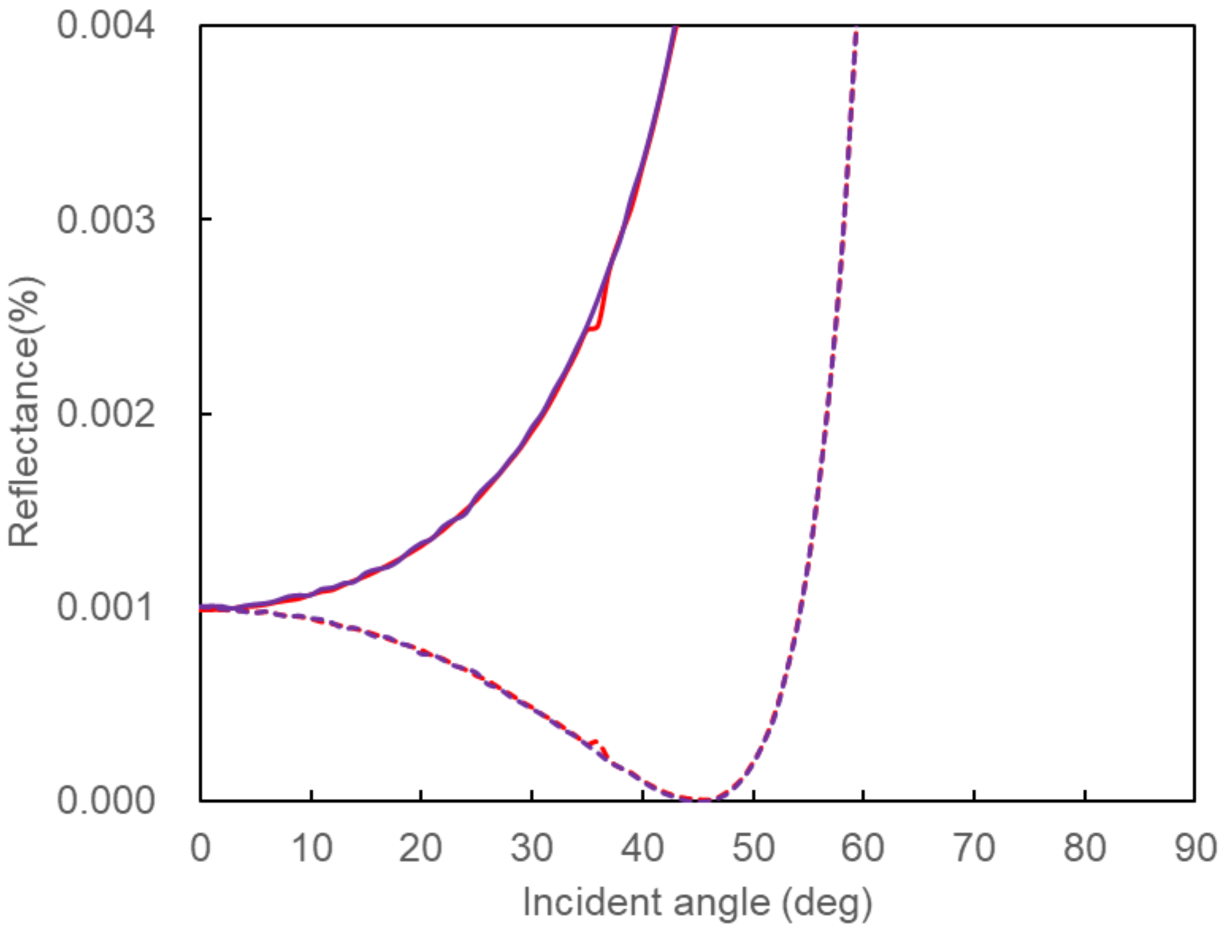

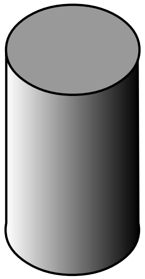

Pillar-model

Solid line: TE wave
Broken line: TM wave
Green: grid pattern
Purple: honeycomb pattern

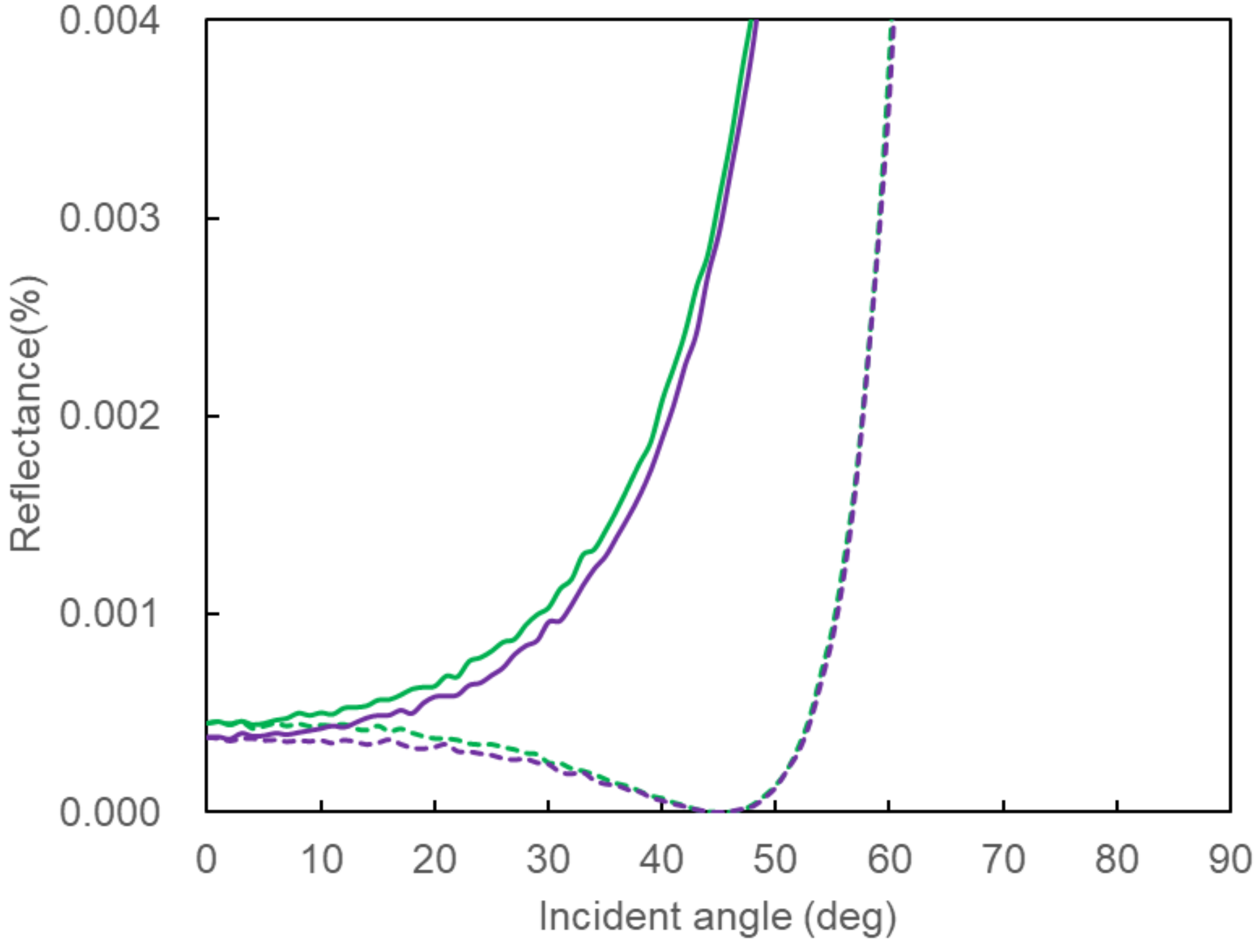

In the pillar model, the reflectance was smaller on the honeycomb pattern than the reflectance on the grid pattern in the most range of the incident angle, and the difference between the patterns was less than 0.04 points for TE wave and less than 0.01 points~~%~~ for TM wave.

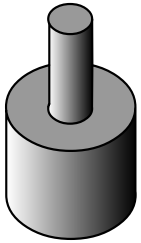

Two-tier

Solid line: TE wave
Broken line: TM wave
Blue: grid pattern
Purple: honeycomb pattern

In the two-tier model, the reflectance of honeycomb pattern was often smaller than the reflectance on the grid pattern, and the difference was less than 0.02 points for TE wave and less than 0.01 points for TM wave.

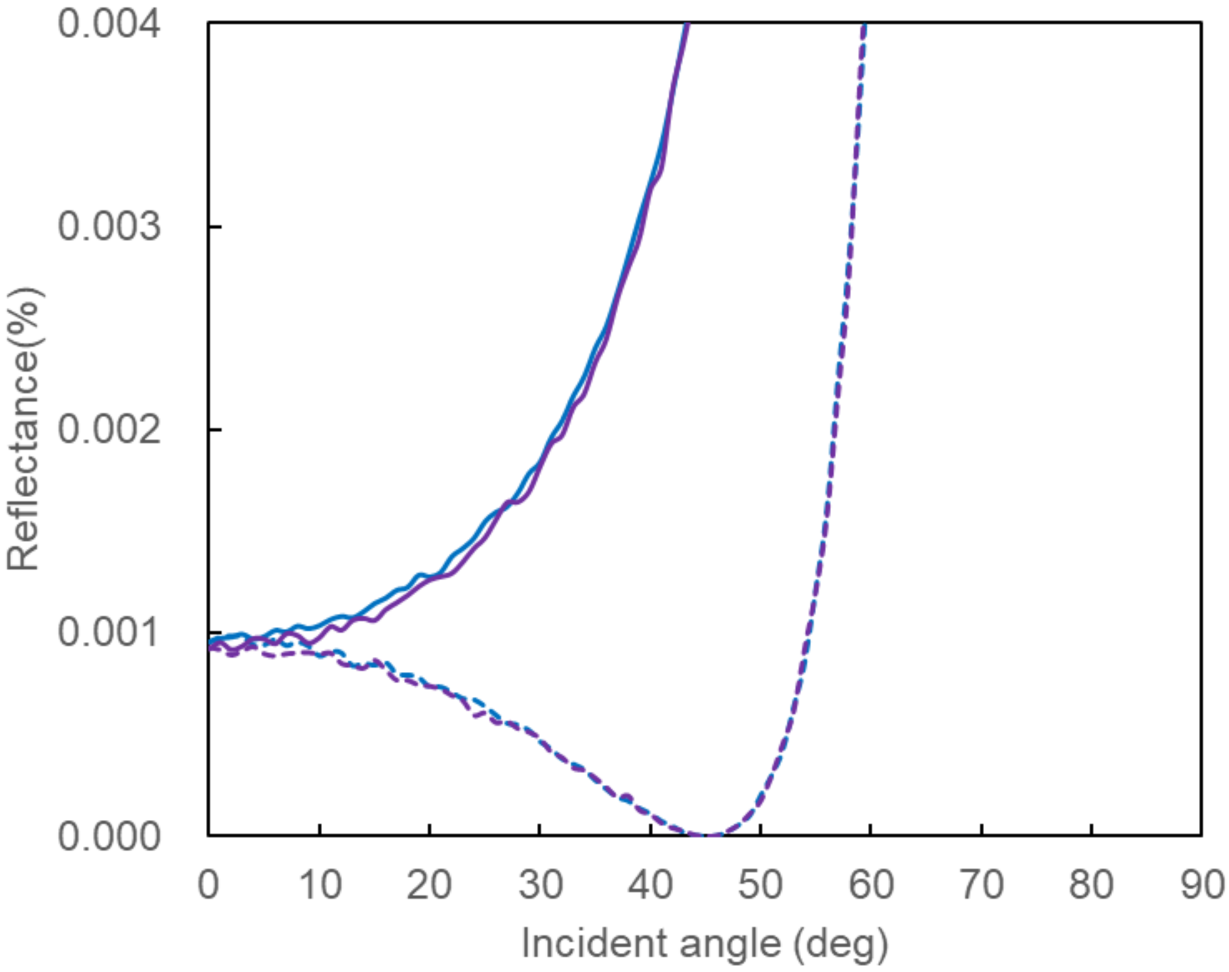

Supplement: Supplementary file 2 — Reflectance of 589-nm light on the nipple array models arranged in the grid pattern and honeycomb pattern. (PDF 252 kb) [file 40851_2018_91_MOESM2_ESM.pdf]
