# Supplementary material for: Physical properties of the tunic in the pinkish-brown salp Pegea confoederata (Tunicata: Thaliacea)
Source: Zoological Lett. 2018 Apr 12;4:7. doi: 10.1186/s40851-018-0091-1 (PMC5896079; doi:10.1186/s40851-018-0091-1)

Supplementary Figure S3

TITLE: Physical properties of the tunic in the pinkish-brown salp *Pegea confoederata* (Tunicata: Thaliacea)
AUTHOR: Daisuke Sakai, Hiroshi Kakiuchida, Jun Nishikawa & Euichi Hirose

Offset of larger reflectance at shorter wavelength by greater anti-reflection effect of nipple array

The difference in refractive indices (Δn) between tunic and seawater is larger when the wavelength of light is shorter, resulting larger reflectance. When the Δn is 0.01 at 589 nm and 0.015 at 400 nm, the reflectance at 400 nm (thick, black lines) is considerably larger than the reflectance at 589 nm on the flat surface (thin, black lines). On the other hand, the reflectance on the nipple array (pillar, honeycomb) at 400 nm (thick, red line) is similar to the reflectance at 589 nm on the flat surface (thin, red line).

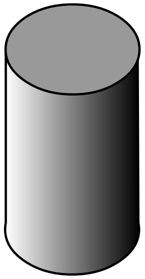

Pillar in honeycomb pattern

Thick line: 400 nm, Δn 0.015
Thin line: 589 nm, Δn 0.01
Black: Flat surface
Red: Nipple array (pillar)

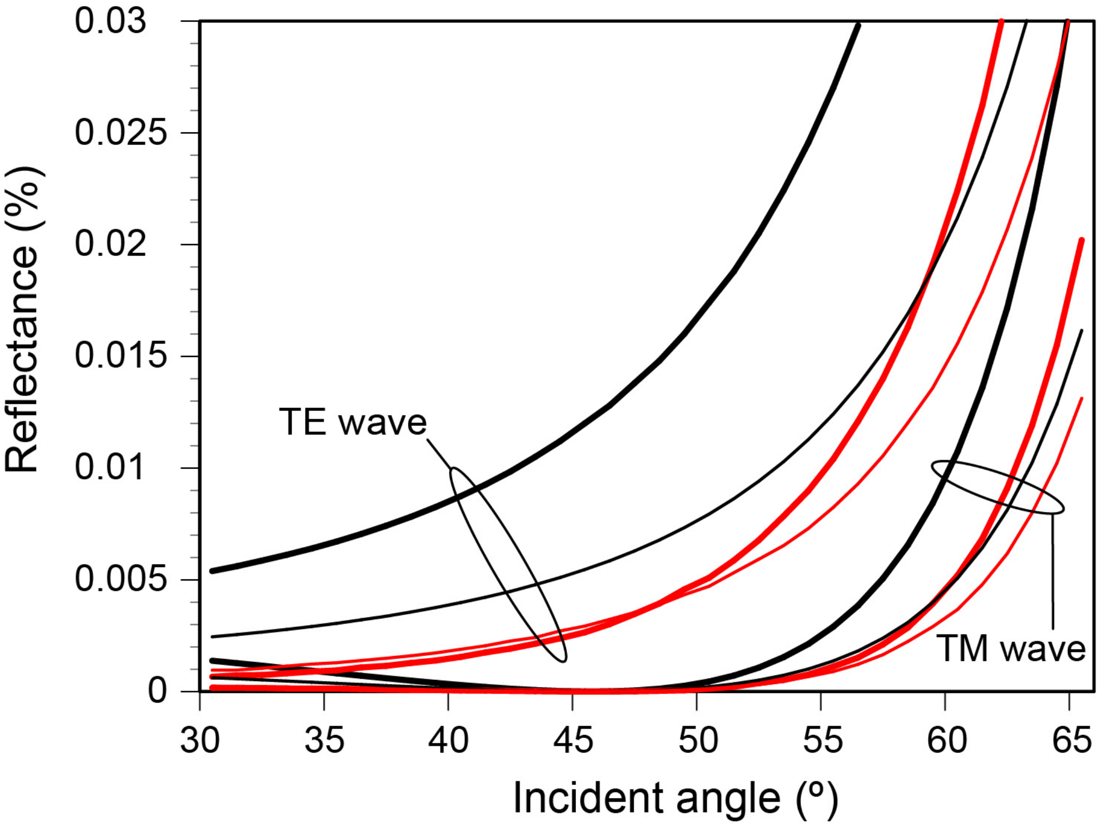

Supplement: Supplementary file 3 — Offset of larger reflectance at shorter wavelength by greater anti-reflective effect of the nipple array. (PDF 395 kb) [file 40851_2018_91_MOESM3_ESM.pdf]
